# Simultaneous induction of jasmonic acid and disease-responsive genes signifies tolerance of American elm to Dutch elm disease

**DOI:** 10.1038/srep21934

**Published:** 2016-02-23

**Authors:** S. M. Sherif, M. R. Shukla, S. J. Murch, L. Bernier, P. K. Saxena

**Affiliations:** 1 Gosling Research Institute for Plant Preservation, Department of Plant Agriculture, University of Guelph, Guelph, ON, Canada; 2 Department of Horticulture, Faculty of Agriculture, Damanhour University, Al-Gomhuria St., PO Box 22516, Damanhour, Al-Behira, Egypt; 3 Chemistry Department, University of British Columbia, Kelowna, BC, Canada; 4 Centre d’étude de la forêt (CEF) and Institut de biologie intégrative et des systèmes (IBIS), Université Laval, Québec City, QC, Canada

## Abstract

Dutch elm disease (DED), caused by three fungal species in the genus *Ophiostoma*, is the most devastating disease of both native European and North American elm trees. Although many tolerant cultivars have been identified and released, the tolerance mechanisms are not well understood and true resistance has not yet been achieved. Here we show that the expression of disease-responsive genes in reactions leading to tolerance or susceptibility is significantly differentiated within the first 144 hours post-inoculation (hpi). Analysis of the levels of endogenous plant defense molecules such as jasmonic acid (JA) and salicylic acid (SA) in tolerant and susceptible American elm saplings suggested SA and methyl-jasmonate as potential defense response elicitors, which was further confirmed by field observations. However, the tolerant phenotype can be best characterized by a concurrent induction of JA and disease-responsive genes at 96 hpi. Molecular investigations indicated that the expression of fungal genes (i.e. *cerato ulmin*) was also modulated by endogenous SA and JA and this response was unique among aggressive and non-aggressive fungal strains. The present study not only provides better understanding of tolerance mechanisms to DED, but also represents a first, verified template for examining simultaneous transcriptomic changes during American elm-fungus interactions.

Over the last century, American elms (*Ulmus americana* Planch) have disappeared from streets of many North American towns and cities. It has been estimated that by the 1980 s, nearly 60% of the 77 million American elm trees in the USA were destroyed by Dutch elm disease (DED)^1^. The disease is caused by three fungal species; the most aggressive among them is *Ophiostoma novo-ulmi* Brasier^2^ which is responsible for the last, current pandemic. The other, less-aggressive species is *O. ulmi* (Buism.) Nannf^3^. which caused the first pandemic that spread through Europe from the early 1900’s to the 1940’s^4^. The third species, *O. himal-ulmi*, is usually endemic to the western part of Himalayas and despite being an endophyte of native elms, it caused typical DED symptoms when introduced to European elm species and hybrids by controlled inoculations^5^. DED fungi are delivered by the vector elm bark beetles from the genera *Scolytus* (*S. scolytus, S. laevis*, *S. kirchi, S. pygmaeus*, *S. multistriatus*, *S. schevyrewi*) and *Hylurgopinus* (*H. rufipes*)^6^,^7^,^8^. Beetles carry fungal spores (conidia) on the surface of their bodies and in the gut. The fungal spores are injected into the elm xylem tissues during beetle tunneling and feeding. The elms produce tyloses and gels as defense mechanisms but these, along with the insects and growing fungi obstruct the tree vasculature and reduce water uptake. The host tree wilts and eventually dies as a result of both the infestation and the infection.

Management of DED includes sanitation and the use of registered pesticides and fungicides but has had limited success. A number of American elms have escaped the last pandemic and may be showing systemic tolerance to DED. It is estimated that 1 in 100,000 *U. americana* may be tolerant to DED^9^. Breeding programs have successfully selected and released cultivars such as ‘Valley Forge,’ ‘Princeton,’ ‘Delaware,’ and ‘New Harmony,’ that are tolerant to DED^10^,^11^. Although the planting of tolerant trees would be a significant advancement in sustainable management, the existing cultivars have limited distribution, the tolerance mechanism is not well understood and true resistance has not been achieved. Attempts to generate resistant cultivars through crossbreeding with closely related resistant *Ulmus* species (i.e. *U. pumila* and *U. parvifolia*) have generally failed due to sexual incompatibility^12^,^13^,^14^. Protoplast fusion techniques and somatic hybridization are new approaches to overcome these problems. Recently, a protocol for regeneration of plants from cell suspension-derived protoplasts was developed^15^ making such experiments possible. Identifying the specific mechanisms of resistance to DED is the first step in cellular breeding of resistant cultivars.

Plants respond to microbes by producing many defense-related compounds, most of them being inducible. Plant signaling pathways mediated by salicylic acid (SA) and jasmonic acid (JA) are thought to form the backbone of the plant defense system, and the reactions associated with other plant hormones might be ultimately a result of cross-talks with this SA-JA backbone^16^. It is generally well-established, with some exceptions, that SA confers resistance against biotrophs, which obtain their nutrients from living cells, whereas JA confers resistance against necrotrophs, which destroy host cells, often through cell-wall-degrading enzymes and phytotoxins, then feed on their contents^16^,^17^. Unfortunately, the lifestyle of DED fungi and whether they should be considered as biotrophs, necrotrophs or hemi-biotrophs is still uncertain^18^,^19^,^20^. In addition, the application of SA, JA or their derivatives as defense elicitors to enhance resistance to DED has yielded contrasting results^19^,^20^,^21^. Given that the efficacy of these defense elicitors has been examined under different conditions and using different elm species and genotypes, it is thought that applying SA and JA under the same experimental settings and using genotypes with known responses to DED would eradicate such existing contradiction.

The different levels of foliar symptoms and crown dieback observed among tolerant trees suggest that multiple genes are involved in conferring tolerance and resistance to DED^22^,^23^ but very little is known about the regulation and induction of tolerance to DED at the molecular level. Only one large-scale cDNA sequencing approach was used to identify genes upregulated in American elm calli after compatible interactions with an aggressive strain of *O. novo-ulmi*^24^. Despite the significance of this study in providing, for the first time, a valuable list of American elm genes associated with DED disease, the differential regulation of these genes between the tolerant and susceptible genotypes has never been investigated. In addition, the expression of genes in undifferentiated cells (i.e. callus tissues) cannot truly reflect the scenario in xylem tissues where the natural infection takes place. Indeed, in a separate study to compare the differential expression of defense-related genes encoding phenylalanine ammonia-lyase (PAL), chitinase (CHT) and polygalacturonase-inhibiting protein (PGIP) between the DED-resistant *U. pumila* and DED-susceptible *U. americana*, researchers demonstrated tissue-specific expression of these genes, especially *CHT* which was hardly detectable in tissues derived from callus, while showing high expression in roots and leaf midribs^25^. Although callus tissues are generally more amenable to transcriptomic analysis than woody stems^26^, the elm’s vascular defense responses to DED could be different.

A suppression subtractive hybridization library constructed from American elm calli inoculated with *O. novo-ulmi* has resulted in a list of 53 genes (collectively referred to herein as disease-responsive genes) differentially upregulated in inoculated vs. the mock-inoculated calli^24^. Among these, genes with high copy numbers were used in the present study to compare between compatible and incompatible American elm – *O. novo-ulmi* interactions at the molecular level, using four-year-old tolerant and susceptible American elm saplings as a template. Our experiments provided novel insights into the molecular detection of fungal infection of American elm tissues and led to the development of an efficient method for nucleic acid extraction from woody elm stems. These findings, together with the analyses of endogenous JA and SA in tolerant and susceptible elm saplings, and the efficacy of SA and methyl-jasmonate (MeJA) as defense elicitors in the field, will facilitate further investigation of the mechanisms of tolerance and susceptibility of American elm to DED.

## Results

### Molecular detection of fungal genes in infected elm stems

**Figure 1:**
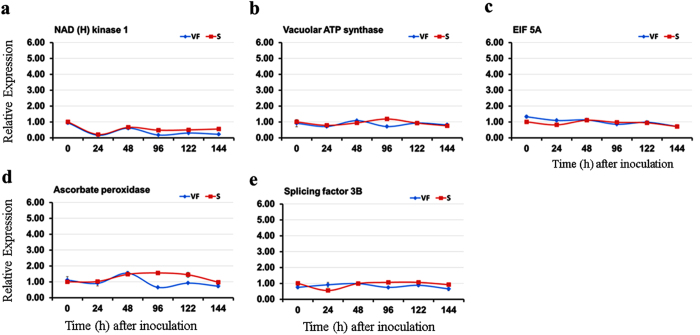
The transcriptional stability of five internal reference genes in infected and non-infected elm saplings. (**a–e**) The expression of five reference genes encoding NAD (H) kinase 1, vacuolar ATP synthase, EIF 5a, ascorbate peroxidase and splicing factor 3B was investigated in saplings of ‘Valley Forge’ (VF) and the susceptible (S) elm clone at 0–144 hpi with the *O. novo-ulmi* fungus. The 72 hour sampling time point was missing due to uncontrollable circumstances. The expression of each gene was calculated relative to the control sample (0 hpi) in the susceptible clone.

Most approaches employed to assess the sensitivity to DED are based on visual measurements of disease symptoms (i.e, vascular discoloration, crown dieback), which despite their ease, inherently include multiple variables that lead to false positives. Molecular techniques based on the expression of fungal genes by qRT-PCR can lead to more specific and quantitative results. To examine the efficacy of these approaches for DED detection, a DED-tolerant cultivar ‘Valley Forge’ and a susceptible American elm clone were evaluated. In addition, a modified CTAB method was used for RNA extraction from the woody tissues of elm stems. To normalize the gene expression data, the induction kinetics of five internal reference genes were investigated in control (0 hpi) and infected stems of the tolerant and susceptible elm saplings. These genes encode eukaryotic translation initiation factor 5 A (EIF 5a), NAD (H) kinase 1, vacuolar ATP synthase, ascorbate peroxidase and splicing factor 3B. Although these genes have shown stable transcription rate when examined in control and infected elm calli^24^, only three of them (*EIF 5a*, *vacuolar ATP synthase* and *splicing factor 3B*) exhibited transcriptional stability in elm stems in various conditions, and a high level of amplification specificity, as illustrated by the dissociation curve (Fig. 1 and Supplementary Fig. 1).

**Figure 2:**
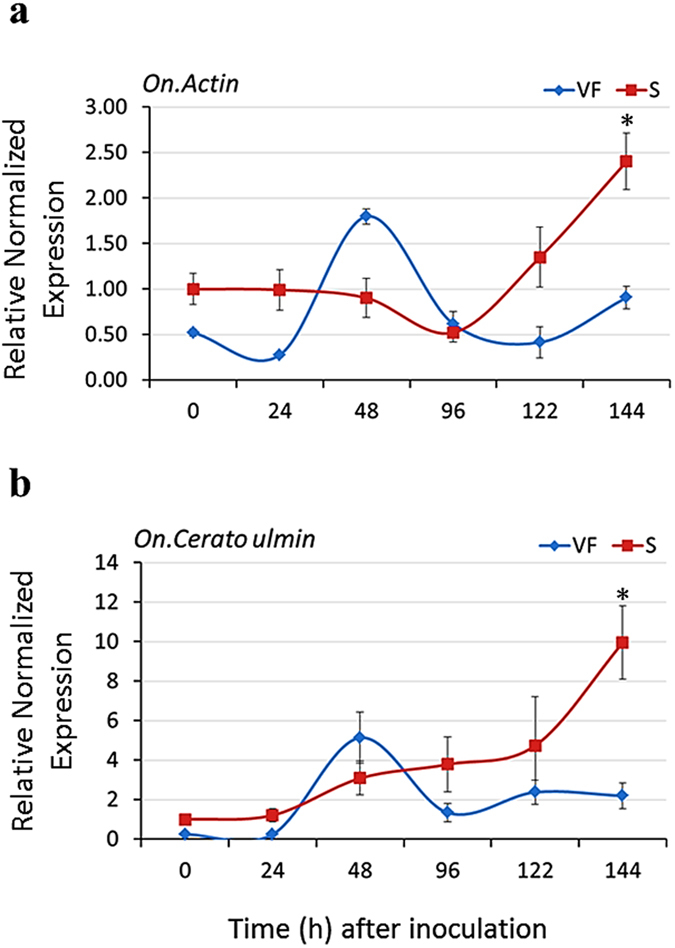
Expression of fungal genes in infected elm wood tissues. **(a,b)** induction kinetics of fungal *On-Actin* and *On-Cerato ulmin* genes in American elm wood tissues infected with *O. novo-ulmi* fungus. Four-year-old saplings of ‘Valley Forge’ (VF) and the susceptible clone (S) were infected with the fungus and 2 cm^2^ of tissues surrounding the infection zone were collected at 0–144 hpi. The expression of each gene was normalized to that of three reference genes (*EIF 5a*, *vacuolar ATP synthase* and *splicing factor 3B*) and was calculated relative to the control sample (0 hpi) in the susceptible clone. Values represent the mean ( ± SE) of three biological replicates. Values marked with an asterisk (*) are significantly greater (≥2 times) than the corresponding time point in the other genotype (*P* < 0.05).

The expression of *O. novo-ulmi* genes (*On*-*Actin* & *On*-*CU*) was quantified in four-year-old saplings of the tolerant and susceptible elm genotypes infected with the fungus (MH75-4O) for 144 h. When the expression of fungal genes (*On*-*Actin* & *On*-*CU*) was normalized to that of three elm reference genes (*EIF 5a*, *vacuolar ATP synthase* and *splicing factor 3B*), transcripts from both fungal genes were more readily detected in the susceptible genotype at 144 hpi. No significant difference was observed between elm genotypes at the first 122 h of inoculation (Fig. 2a,b). The simultaneous detection and PCR-amplification of American elm and fungal genes in the infected elm specimens was effective as indicated by the comparable threshold cycle (Ct) values of the studied genes (Supplementary Fig. 3a). In order to examine the specificity of primers used, the dissociation curve (melt curve) was obtained for each PCR reaction. The presence of a single peak (one melting point) points to a single amplicon, confirming the specificity of primers and the consistency of the qRT-PCR results (Supplementary Fig. 3b). Together, these results demonstrate the validity of the modified CTAB method, the reliability of the qRT-PCR approach in the detection of *O. novo-ulmi* in infected elm specimens and the tolerance of ‘Valley Forge’ to DED.

### Microscopic detection of the fungus in infected elm stems

**Figure 3:**
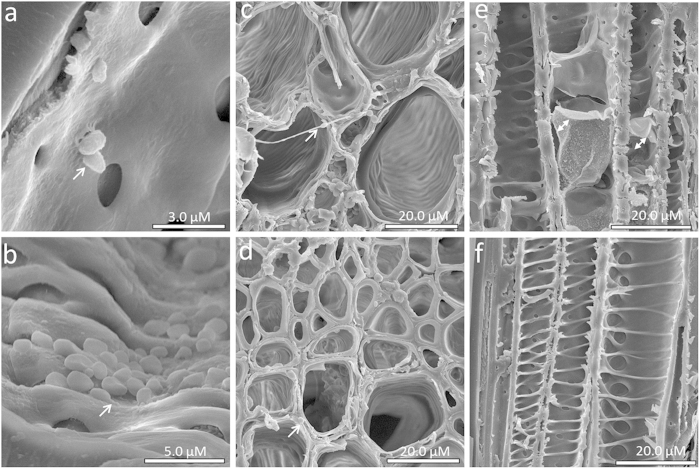
SEM observations of *O. novo-ulmi* growth and elm defense responses within 144 hpi. (**a)** a xylem vessel of the susceptible clone at 48 hpi. Arrow refers to conidia attached to xylem vessel wall. (**b)** Germinating conidia observed inside a xylem vessel of the susceptible clone at 96 hpi. Arrow refers to a germ tube. (**c,d**) cross sections in the stem of the susceptible clone and ‘Valley Forge’ at 96 hpi, respectively. Arrows refer to a developing hypha and germinating conidium, respectively (**e,f)** a longitudinal section in the stem of VF at 144 hpi. Double-headed arrows refer to tyloses inside xylem vessels. All images were obtained from the xylem elements in the two most recent annual growth rings of the sapwood.

The aggressiveness of MH75-4 O strain was verified in mature American elm trees (≥10-year-old) and the typical disease symptoms were conspicuous within 2-weeks of inoculation (Supplementary Fig. 2). However, the infected saplings of the tolerant and susceptible genotypes did not show any visible disease symptoms along the experimental period (144 hpi). To further validate the results obtained by qRT-PCR for fungal growth over the first 144 hpi, the infected stems of the susceptible and tolerant elm genotypes were examined by scanning electron microscopy (SEM). In general, the detection of fungal spores and hyphae was much easier in the susceptible genotype. At 48 hpi, only conidia were detected inside xylem vessels, attached to the vessel cell wall; but no direct penetration of vessel cell wall or xylem pits was detected in the specimens examined (Fig. 3a). At 96 hpi, different stages of fungal development were observed, especially in the susceptible genotypes where many conidia and hyphae were found inside or among xylem elements, respectively (Fig. 3b–d). No developing hyphae could be detected in the tolerant ‘Valley Forge’ at this stage. Plant defense responses represented by tyloses in the vessel lumen were more evident at 144 hpi in both genotypes (Fig. 3e). However, tyloses were not prevailing in all vessels (Fig. 3f), which could explain the absence of typical leaf wilting symptoms associated with DED during the time-frame of the experiment.

### Differential expression of disease-responsive genes in tolerant and susceptible genotypes

**Figure 4:**
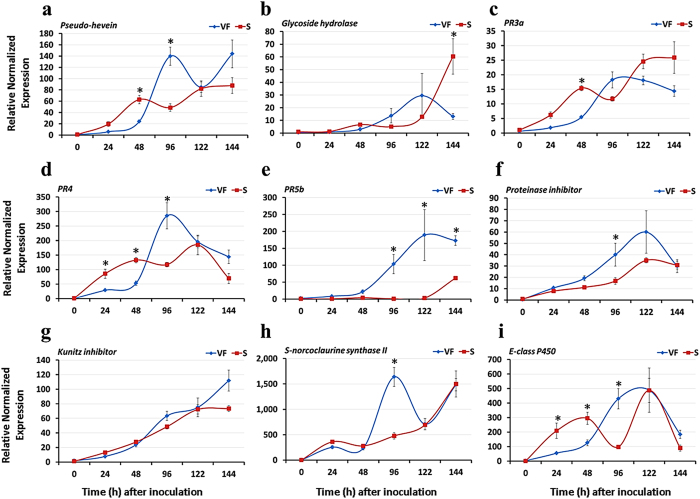
Differential expression of disease-responsive genes in tolerant and susceptible American elm saplings. **(a–i)** The expression of nine genes encoding different classes of disease-responsive proteins was investigated in saplings of ‘Valley Forge’ (VF) and the susceptible (S) elm clone at 0–144 hpi with the *O. novo-ulmi* fungus. The expression of each gene was normalized to that of three reference genes (*EIF 5a*, *vacuolar ATP synthase* and *splicing factor 3B*) and was calculated relative to the control sample (0 hpi) in the susceptible clone. The results are the mean ± SE of three biological replicates. Values marked with an asterisk (*) are significantly greater ( ≥ 2 times) than the control sample and the corresponding time point in the other genotype (*P* < 0.05).

The induction kinetics of the disease-responsive genes was examined in the infected saplings of ‘Valley Forge’ and the susceptible clone at different times after inoculation with *O. novo-ulmi* (MH75-4 O). In the susceptible clone, nine out of the fifteen genes analyzed showed the same expression pattern observed in infected elm callus tissues^24^, in which transcript levels showed steady increase over time and reached to maximum levels at 122 or 144 hpi (Fig. 4 and Supplementary Fig. 4). However, there was a noticeable decline in transcript levels at 96 hpi for most genes tested, which was not the case with the infected elm calli^24^, suggesting that systemic responses in intact elm stems might alter the local cellular reactions to fungal infection. On the other hand, the expression of genes in infected saplings of ‘Valley Forge’ showed a unique induction pattern, in which gene expression increased slightly at the first 48 hpi and then showed a strong induction peak at 96 or 122 hpi. Genes encoding pathogenesis-related proteins (i.e *PR4*, *PR5b*), proteinase inhibitors, pseudo-hevein and E-class P450 showed ≥ 2-fold higher expression in ‘Valley Forge’ than the susceptible clone at 96 hpi (Fig. 4a,d,e,f,h,i). Most of the studied genes showed upregulation in the susceptible genotype in at least one single point post-inoculation, collectively suggesting that differences between tolerance and sensitivity to DED are not necessarily based on presence or absence of certain disease-responsive genes, respectively, rather they are established by the timing and level of the gene expression in the genotypes.

### Phenylpropanoid pathway-related genes and compounds in infected elm stems

**Figure 5:**
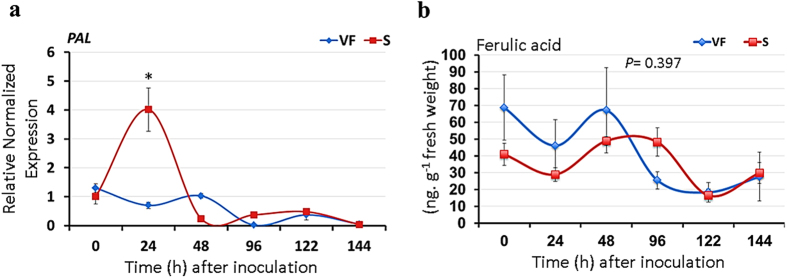
Induction profiles of phenylpropanoid pathway-related genes and compounds in tolerant and susceptible elm saplings. (**a**) Expression of the gene encoding phenylalanine ammonia-lyase (PAL) was monitored in stems of ‘Valley Forge’ and the susceptible clone after different times of inoculation with *O. novo-ulmi*. The expression of *PAL* was normalized to that of three reference genes (*EIF 5a*, *vacuolar ATP synthase* and *splicing factor 3B*) and was calculated relative to the control sample (0 hpi) in the susceptible clone. Values marked with an asterisk (*) are significantly greater (≥2 times) than the control and the corresponding time point in the other genotype. (**b)** the level of ferulic acid (ng g^−1^ fresh weight) was quantified in stems of ‘Valley Forge’ and the susceptible clone after different times of inoculation with *O. novo-ulmi*. The results are the mean ± SE of three biological replicates.

A group of genes involved in phenylpropanoid pathway are differentially expressed in inoculated elm callus^24^, which with other findings^18^, led the authors to suggest that phenolic compounds may play a significant role in tolerance to DED. To examine this notion in American elm saplings, two genes (*isoflavone reductase*, *O-methyltransferase*) encoding enzymes in this pathway were investigated in tolerant and susceptible saplings, but these showed no distinct expression patterns between compatible and incompatible interactions (Supplementary Fig. 4). It is noteworthy that the expression of *isoflavone reductase* increased post-inoculation over time and was generally higher in the susceptible clone, which is in agreement with the high representation of this gene in inoculated callus tissues^24^. The gene encoding phenylalanine ammonia lyase (PAL) did not also show any significant difference between ‘Valley Forge’ and the susceptible clone at most sampling times post inoculation. Surprisingly, *PAL* was upregulated to a significantly higher level in the susceptible clone only at 24 hpi (Fig. 5a). Moreover, the production kinetics of ferulic acid, one of the known phenylpropanoid compounds implicated in resistance to pathogenic fungi^27^,^28^,^29^, showed no significant difference (*P* = 0.397) between the tolerant and susceptible genotypes after inoculation (Fig. 5b), indicating again that the callus tissues might show unique responses to fungal invasion that are not necessarily similar to the vascular defense responses.

### Levels of salicylic acid (SA) and jasmonic acid (JA) in infected American elm saplings

**Figure 6:**
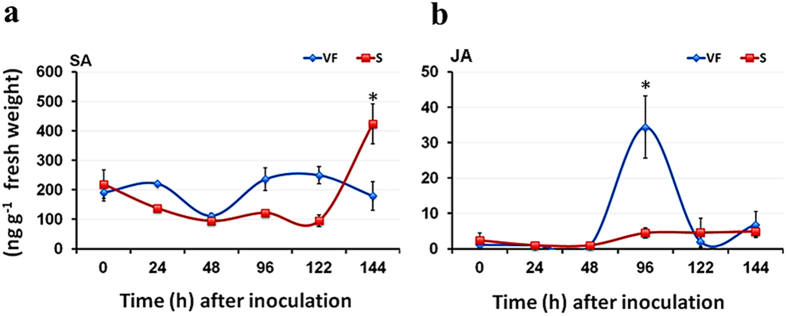
Levels of salicylic acid (SA) and jasmonic acid (JA) in infected Valley Forge (VF) and susceptible (S) American elm saplings. (**a,b**) The levels of SA and JA were assessed in stem tissues (~2 cm^2^) around the inoculation zone at 0, 24, 48, 96, 122 and 144 hpi with the *O. novo-ulmi* (10^7^ spores/ml). The results are the mean ± SE of three biological replicates. Values marked with an asterisk (*) are significantly higher (*P* < 0.05) than the control sample (0 hpi) and the corresponding time point in the other genotype.

It has become evident that SA and JA represent the backbone of the plant defense system^16^. To determine which pathway is involved in the differential expression of disease-responsive genes between the tolerant ‘Valley Forge’ and the susceptible elm genotypes, the levels of these plant hormones were quantified in their saplings after different times of inoculation. The results generally show that SA levels in both the tolerant and susceptible genotypes were similar (~200 ng g^−1^ fresh weigh) at 0 hpi, but a significant reduction (*P* < 0.05) was noticed in the susceptible genotype within the first 122 hpi. On the other hand, the level of SA in the tolerant genotype was significantly unchanged after inoculation, except at 48 hpi. The induction of SA in the susceptible genotype was observed only in tissues sampled at a later time point (144 hpi) (Fig. 6a). Unlike SA, JA showed only one induction peak in ‘Valley Forge’ at 96 hpi and no significant increase in JA levels was noticed in the susceptible genotype at any time post-inoculation (Fig. 6b). These results indicate that the higher expression of disease-responsive genes noticed in ‘Valley Forge’ at 96 hpi could be regulated mostly by JA, which was detected at high levels during this time.

### Most of disease-responsive genes are upregulated by JA

**Figure 7:**
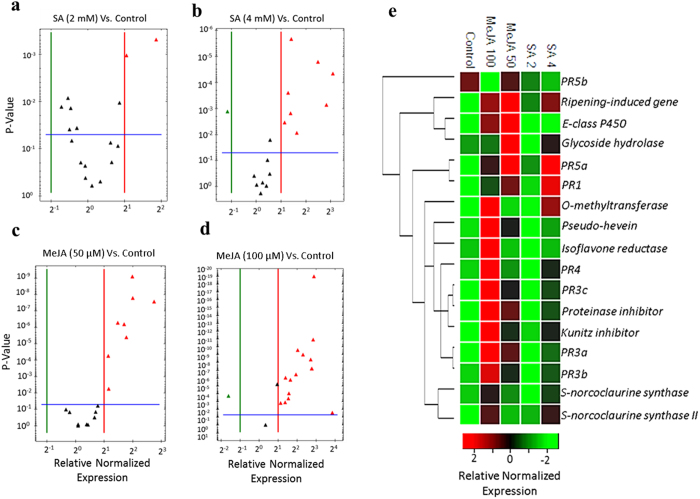
Gene expression analysis of disease-responsive genes after treatments with SA or MeJA. (**a–d**) Volcano plots showing the magnitude of differential expression between control (0.01% ethanol) and treatments with SA (2 mM), SA (4 mM), MeJA (50 μM) or MeJA (100 μM). The horizontal line marks the threshold (*P* < 0.05) for defining a gene as up-regulated in control (black) or treatment (red), with a combined change > 2-fold. **(e)** A clustergram with a red/black/green color scheme to show genes where the expression upregulated, unchanged or down regulated, respectively, compared to control.

In order to examine whether the exogenous application of defense elicitors (SA & MeJA) can induce the expression of disease-responsive genes, saplings of the susceptible genotype were sprayed with aqueous solutions containing 0.01% ethanol (control), SA (2 mM & 4 mM) or MeJA (50 μM & 100 μM). The expression of disease inducible genes was quantified using qRT-PCR and transcript levels were normalized to those of three reference genes (*EIF 5a*, *vacuolar ATP synthase* and *splicing factor 3B*), which showed transcriptional stability after treatment with different elicitors (Supplementary Fig. 5). The presentation of results in volcano plots clearly showed that most of the disease-responsive genes are significantly (*P* < 0.05) induced by the highest concentration of MeJA (100 μM), followed by MeJA (50 μM). The application of SA at high concentration (4 mM) also induced the expression of many genes compared to control. A few genes were upregulated by the application of the lowest SA concentration (2 mM) (Fig. 7a–d). It is also worth noting that many genes showing higher expression in the tolerant genotype (i.e. *PR4*, *kunitz inhibitor*, *pseudo-hevein*, *proteinase inhibitor*, *E-class P450*) are also induced by the exogenous application of MeJA (Fig. 7e and Supplementary Fig. 6). However, the induction of these genes by fungal infection was many times higher than defense elicitors (SA or MeJA), indicating that signaling pathways other than SA and JA might also be involved in mediating tolerance mechanisms.

### The application of defense elicitors enhances the field tolerance to DED

**Figure 8:**
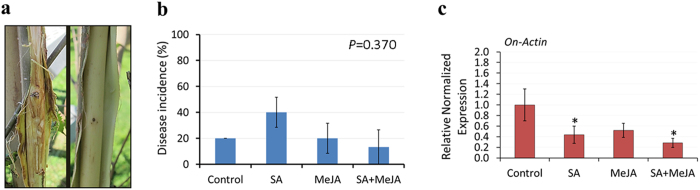
Assessment of disease incidence in American elm seedlings infected with the fungus and treated with defense elicitors. **(a)** Images showing the disease symptoms of Dutch elm disease as brown streaking on the wood of infected American elm seedlings (left) versus tissues without apparent disease symptoms (right). **(b)** The rate of disease incidence was evaluated based on the occurrence of brown streaking on wood tissues (80 cm above the inoculation point) two months after inoculation with *O. novo-ulmi* (10^7^spores/ml). Four-year-old American elm seedlings were treated with 0.01% ethanol (Control), SA (2 mM) at the 1^st^ day of inoculation, MeJA (100 μM) at 1^st^ day of inoculation or SA (2 mM) at 1^st^ day followed by MeJA (100 μM) at the 4^th^ day of inoculation. **(c)** Stem tissues (80 cm above the inoculation point) were collected from control and treated seedlings at 60 days post-inoculation, frozen in liquid nitrogen and then used for quantifying the expression of fungal actin (*On-Actin*). The expression of *On-Actin* was normalized to that of three reference genes (*EIF 5a*, *vacuolar ATP synthase* and *splicing factor 3B*) and was calculated relative to the control sample. Values marked with an asterisk (*) are significantly lower (*P* < 0.05) than control.

The observation that defense elicitors increase the expression of disease-responsive genes led us to examine the effectiveness of applying these molecules as a disease management strategy in the field. Four-year-old American elm seedlings were obtained from a commercial nursery, inoculated with *O. novo-ulmi* (MH75-4 O) and then exposed to treatments by SA (2 mM), MeJA (100 μM) or a combined application of SA and MeJA. SA was applied at the 1^st^ day of inoculation, MeJA was applied at the 1^st^ day of inoculation, and the combined application was applied as SA at the 1^st^ day followed by MeJA at the 4^th^ day. Treated and control American elm seedlings were organized in the field in triplicates (n = 5 seedlings) and maintained for 60 days before recording the rate of disease incidence (%). Interestingly, most of the seedlings showed typical symptoms of DED including leaf browning, wilting, and even death of some lateral branches, but no complete death was observed in any of the inoculated seedlings. The assessment of disease incidence based of the percentage of seedlings showing brown streaking on the wood tissues indicated no significant difference (*P* = 0.370) after treatments with defense elicitors compared to control (Fig. 8a,b). On the other hand, when the qRT-PCR approach was employed to quantify the expression of the fungus *On*-*Actin* gene in the wood tissues 80 cm above the inoculation point, results demonstrated that the application of SA alone or in combination with MeJA significantly (*P* < 0.05) reduced the expression of *On-Actin* compared to control treatment. The application of MeJA at the first day of inoculation reduced the expression of *On-Actin*, but was not significantly different than control (Fig. 8c). These results confirm again that detection approaches based on molecular techniques can lead to early detection and more consistent results. In addition, these results indicate that the application of SA can enhance field tolerance to DED.

### The expression of cerato-ulmin is highly repressed by SA

**Figure 9:**
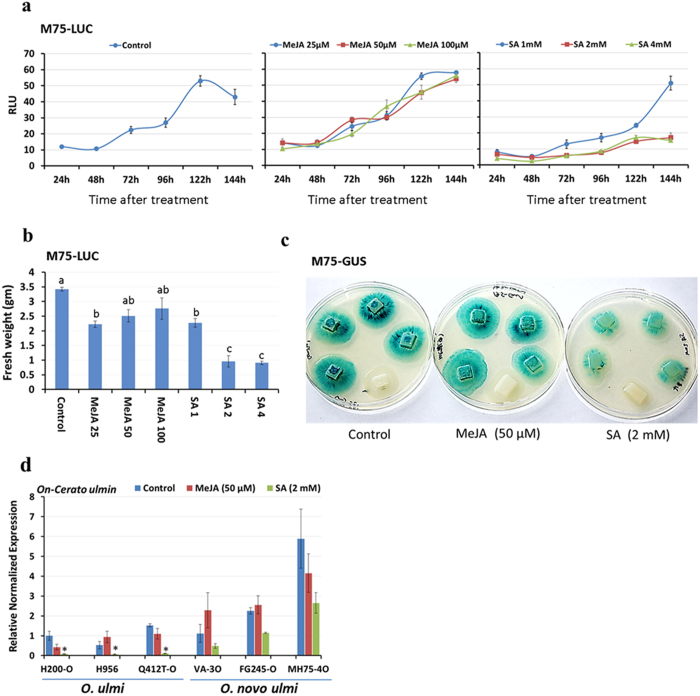
The differential expression of *cerato ulmin* gene after treatments with plant defense elicitors using M75-LUC and M75-GUS reporter systems. (**a**) M75-LUC (L8) transgenic fungal strain was used to observe the induction kinetics of *cerato ulmin* gene in liquid media treated with 0.01% ethanol (Control), SA (1, 2, 4 mM) or MeJA (25, 50, 100 μM). The relative luminescence units (RLU) were recorded for each sample every 24 h and for 144 h after treatment. Values represent the mean ( ± SE) of three biological replicates. (**b**) The fresh weight (gm) of fungal pellet was determined after treatment of fungal liquid media with 0.01% ethanol (Control), SA (1, 2, 4 mM) or MeJA (25, 50, 100 μM) for 144 h. Values marked with the same letter are not significantly different (*P* < 0.05). (**c**) M75-GUS (L4) transgenic fungal strain was used to observe the differential expression of *cerato ulmin* in solid media treated with 5 ml of 0.01% ethanol, SA (2 mM) or MeAJ (50 μM) for 24 h and then stained with GUS staining solution for 24 h at 37 °C. Each plate contains four mycelial plugs of (M75-GUS) strain and one mycelial plug of (M75-LUC) strain (negative control). (**d**) The expression of *On-Cerato-ulmin* gene was quantified in fungal tissues grown in liquid medium treated with 0.01% ethanol (Control), SA (2 mM) or MeJA (50 μM) for 122 h. The expression of *On-Cerato-ulmin* was examined in three strains of *O. ulmi* (H200-O, H956, Q412T-O) and *O. novo-ulmi* (VA-3 O, FG245-O, MH75-4 O). Values represent the mean (±SE) of four biological replicates. The expression of *On-Cerato-ulmin* was normalized to that of *On-Actin* in the same sample and was calculated relative to the expression of *On-Cerato ulmin* in H200-O control sample. Values marked with an asterisk (*) are significantly lower (≤4-times) compared to the control treatment (*P* < 0.05).

To facilitate molecular and cellular investigations of American elm- *O. novo-ulmi* interactions, reporter gene assays based on *GUS*, *LUC* or *GFP* genes driven by the *cerato ulmin* (*CU)* promoter were established^30^. To examine whether the accumulation of SA and JAs in tolerant elm genotypes can also alter the expression of fungal genes, transgenic fungal strains (M75-LUC and M75-GUS) were exposed to these defense elicitors *in vitro*. Despite the long-standing debate about the role of CU in fungal pathogenicity, the hydrophobic nature of this protein and its existence in the fungal cell wall suggests its role in disease establishment, at least during the early stages of infection^31^. Fungal strains expressing LUC (M75-LUC) and grown in liquid medium containing SA (1, 2, 4 mM), MeJA (25, 50, 100 μM) or 0.01% ethanol (control) showed differential LUC activity after different treatments. As shown in Fig 9a, the LUC activity, expressed as relative luminescence units (RLU), increased over the time in control samples, reaching its maximum level at 122 h of treatment. The application of MeJA at different concentrations did not affect LUC activity compared to control. On the contrary, adding SA to the liquid medium led to substantial reductions in LUC activity compared to control, especially at the higher concentrations (SA 2, 4 mM). The quantification of fungal fresh weight (gm) indicated that fungal growth was significantly reduced by SA (Fig. 9b). To exclude such negative effect of SA on fungal growth, the expression of *CU* promoter-driven GUS was investigated in M75-GUS grown on solid medium for three days and then exposed to 0.01% ethanol (control), MeJA (50 μM) or SA (2 mM) for 24 h. After exposing the plates to GUS staining, the results again confirmed the negative effects of SA on the expression of *CU*. Little difference was noticed between control plates and those treated with MeJA (Fig. 9c).

### Strains of O. novo-ulmi are more tolerant to SA than those of O. ulmi

The severe effect of SA treatment on the expression of *LUC* and *GUS* driven by the *CU* promoter led us to examine how different fungal strains respond to plant defense elicitors. It is generally known that strains of *O. novo-ulmi* are far more aggressive than those of *O. ulmi*. Three fungal strains from each of the two species were grown in liquid medium containing 0.01% ethanol (control), MeJA (50 μM) or SA (2 mM) for 122 h and then examined for the expression of *On*-*CU* gene after the treatments. In agreement with the findings of other studies^2^,^31^,^32^, *O. novo-ulmi* strains generally expressed more *On*-*CU* than those of *O. ulmi* in control treatments (Fig. 9d). No significant difference was observed between control and MeJA treatments in all studied strains. However, the application of SA to the liquid medium led to ≥ 4-fold reduction in the expression of *On*-*CU* compared to control in *O. ulmi* strains only. No significant difference was noticed between control and SA treatments when *O. novo-ulmi* strains were used (Fig. 9d). These results suggest that the high levels of SA in tolerant elm genotypes at the early hours of infection may slow the progress of the fungus by negatively affecting the expression of essential fungal genes (i.e. *On*-*CU*). These differences between *O. ulmi* and *O. novo-ulmi* might start an intriguing line of research about the evolution of relative tolerance to SA in the latter, more aggressive species.

## Discussion

The development of a rapid and efficient protocol for RNA extraction from the woody tissues of elm stems was a major technical outcome of the present study. This approach was not only successful in studying the expression of elm genes in woody specimens, but it also helped in retrieving fungal RNA in sufficient amounts for pursuing expression analysis of fungal genes in inoculated elm stems, which, to the best of our knowledge, is the first report on the molecular detection of *Ophiostoma* species in American elm trees. The efficiency of this approach was examined by using two fungal genes (*On*-*Actin* and *On*-*CU*) which represent housekeeping and inducible genes, respectively. This approach was also applied to quantify the rate of disease incidence in American elm seedlings in the field, which yielded more consistent results compared to a classical approach of disease assessment resulting in high standard deviation values (Fig. 8b). The availability of genomic resources for *O. novo-ulmi*^33^,^34^, along with the RNA extraction approach described and validated in the present study further paves the way for in depth investigations of American elm transcriptome through the next generation sequencing (NGS) technologies. Together, these techniques can eventually lead to simultaneous monitoring of elm and fungal responses during different stages of elm-fungus interactions.

The time-course expression profiles of disease-responsive genes in ‘Valley Forge’ and the susceptible clone indicated that tolerance and sensitivity to DED can be determined during the first 144 h of infection with *O. novo-ulmi*. Reactions leading to tolerance were generally characterized by a peak induction of defense-related genes at 96–122 hpi; whereas reactions leading to susceptibility were characterized by either early induction (24–48 hpi) with low transcript levels or late induction (144 hpi) with high transcript levels. The comparison between compatible and incompatible interactions indicated that PR genes especially those encoding chitinases (PR3), thaumatin-like proteins (PR5), endochitinase (PR4), S-norcoclaurine synthase (PR10), proteinase inhibitor and kunitz inhibitor (PR6) were expressed much higher and/or earlier in the tolerant cultivar (Fig. 4). Most of these genes have shown antifungal activity *in vitro* and enhanced resistance to pathogenic fungi when constitutively overexpressed *in planta*^35^,^36^,^37^,^38^,^39^,^40^,^41^,^42^. The induction of most of these genes at 96 hpi in the tolerant genotype, along with their demonstrated antifungal activity is in agreement with the SEM observations, where no hyphal growth was detected in ‘Valley Forge’ along the experimental period. Even in the susceptible genotype, no hyphae were detected before the 96 hpi time point indicating the crucial role of defense proteins and molecules at this stage. Given the diverse nature of these defense-related proteins, it can be implied that many upstream signaling pathways should have contributed to the tolerance of the tolerant genotype, which in turn suggests that tolerance to DED is a polygenic, rather than a monogenic trait. Thus, including many tolerant accessions and cultivars in breeding programs may be rewarding in developing a more tolerant progeny of American elms. Further, the *in vitro* propagation and genetic transformation of American elm have already been demonstrated^43^,^44^,^45^, which opens the door for using the findings of the present study in enhancing tolerance to DED through genetic modification.

The induction kinetics of defense-related genes and defense hormones in the tolerant American elm cultivar is similar to incompatible interactions involving pathogens with a hemi-biotrophic lifestyle^46^,^47^. For instance, in interactions involving the Fusarium head blight fungus (*Fusarium graminearum)* and a resistant wheat cultivar, it was found that SA accumulates within 6 hpi; whereas JA level is elevated around 12 hpi^48^. This biphasic accumulation pattern of defense hormones coincides with the upregulation of SA defense pathway genes during the earlier hours and the induction of JA defense pathway genes at the later hours of fungal invasion^48^. Plants might modulate the timing of SA/JA responses to allow for complementary rather than antagonistic interactions between these two pathways during the interaction with hemi-biotrophic pathogens. Indeed, in *Arabidopsis*, the concurrent induction of SA and JA signaling promotes disease severity caused by *F. graminearum.* Furthermore, the exposure of *Arabidopsis* plants to MeJA at the early stages of infection with *F. graminearum* increases disease severity, whereas later application of MeJA (12–24 hpi) leads to disease resistance^49^. These findings together lead to a suggestion that *O. novo-ulmi* is a hemi-biotrophic pathogen as previously proposed^20^, but not a biotrophic^19^ or a necrotrophic pathogen^18^ as suggested earlier.

Indeed, this potential hemi-biotrophic lifestyle of *O. novo-ulmi* explains other findings in the literature. For instance, the cell wall degradation of callus tissues and the erosion of xylem vessel walls after *in vitro*^18^ and *in vivo*^50^ interactions with *O. novo-ulmi,* respectively, suggest a necrotrophic phase of the fungus. However, the application of MeJA which was shown to be effective in enhancing resistance of other forest trees (i.e. Norway spruce) against necrotrophic pathogens such as *Ceratocystis polonica*^51^,^52^ and *Pythium ultimum*^53^, was not effective when used with *U. minor* against *O. novo-ulmi*^19^. On the other hand, SA which is known to confer resistance against biotrophs^17^, enhanced the resistance of *U. minor* to *O. novo-ulmi* when applied with the irrigation water^20^. Similarly, the findings of the present study showed that application of SA at the first day of infection or the combined application of SA (at early hours) and MeJA (at late hours) were more effective in reducing fungal progress in American elm seedlings than MeJA application alone (Fig. 8c), indicating all together that the fungus might embrace a biotrophic lifestyle at early stages of colonization. Indeed, our microscopic investigations did not show any signs of vessel wall degradation during the attachment or the germination of the fungal spores at the first 48 hpi in both the tolerant and susceptible elm genotypes (Fig. 3a–d). This is also consistent with SEM observations^54^,^55^,^56^ showing that hyphae penetrate vessel pits within 48 hpi but have never been seen penetrating the cell wall directly during the early hours of infection.

Despite the limited number of reports on *Ulmus*-*Ophiostoma* interactions, all of these studies suggest a paramount role for the phenylpropanoid pathway in resistance against DED, which in general contradicts the findings of the present study. In one study, it has been found that the steady-state expression of *PAL* was higher in a resistant compared to a susceptible elm species^25^. However, it should be noted that the expression of *PAL* was not investigated in elm wood tissues or after the challenge with the fungus. In other studies, it has been stated that genes involved in phenylpropanoid pathway are well represented in callus tissues inoculated with the fungus compared to the mock-inoculated calli, and high amounts of phenolic compounds accumulate in infected calli^18^,^24^. However, in the present study, genes and compounds of the phenylpropanoid pathway did not show differential induction in the tolerant genotype (Fig. 5), but they were generally higher in inoculated compared to control tissues. Indeed, a recent report indicated that inhibition of the phenylpropanoid pathway by 2-aminoindane-2-phosphonic acid, an inhibitor of PAL, reduces browning in American elm callus tissues^57^, indicating that excessive production of phenolic compound is natural in elm calli and may have little to do with disease tolerance. Furthermore, the direct correlation between phenylpropanoid pathway-mediated cell wall reinforcement and disease resistance proved untrue is many cases. For instance, the downregulation of lignin activates defense responses and increases the resistance to the hemibiotroph *Colletotrichum trifolii* in alfalfa (*Medicago sativa* L.)^58^. Similarly, it has been demonstrated that inhibition of callose deposition results in SA-dependent disease resistance^59^.

In the past 50 years, a good part of research on DED was centered on cerato ulmin (CU), a phytotoxic, low molecular weight protein, classified as a class II hydrophobin^31^,^60^,^61^,^62^. In the present work, we studied the expression of *CU* as an example of how endogenous plant defense hormones can alter the expression of fungal genes. Although the role of CU in fungal pathogenicity and virulence is still controversial^31^, its role in increasing the hydrophobic and adherent qualities of the yeast-like cells as well as increasing their resistance to desiccation has been demonstrated^63^. Our results indicated that the expression of *CU* was reduced significantly in fungal cultures exposed to SA, but its expression was not affected significantly by MeJA. Moreover, our findings showed that the non-aggressive fungal strains were more affected by SA than the aggressive ones, which points to the possibility of a selection pressure motivated by tolerance to SA. The tolerance of aggressive fungal strains to plant defense hormones opens a new line of investigation on the evolution of virulence in *Ophiostoma novo-ulmi*.

In conclusion, the findings of the present study indicate that tolerance to DED is characterized by a simultaneous induction of defense-related genes and JA at 96 hpi post-inoculation, which probably coincides with the necrotrophic phase of the fungus. The findings also suggest that the role of phenylpropanoid pathway in resistance against DED is not as paramount as previously revealed using elm calli. Moreover, the results clearly demonstrate that cellular reactions of inoculated elm calli are not comparable to those obtained in elm stems where local and systemic responses take place. The results show that SA application can enhance tolerance to DED in the field, which - with other findings - point to a hemi-biotrophic lifestyle of *O. novo-ulmi*. However, more investigations are required to further confirm this notion. Most importantly, the present study introduces a verified template to monitor transcriptomic changes during *Ulmus* - *Ophiostoma* interactions, first documentation of molecular differences between DED-tolerant and sensitive *U. americana* genotypes and novel insights in fungal responses to plant defense hormones.

## Materials and Methods

### Plant Material

Four-year-old American elm saplings of ‘Valley Forge’ were purchased from a commercial nursery (Connon Nurseries, West Flamborough, ON, Canada). The susceptible elm plant material was selected from clones of a susceptible elm tree in the *in vitro* germplasm bank^43^ at the Gosling Research Institute for Plant Preservation (GRIPP), University of Guelph.

### Inoculant

Yeast-like spore suspensions of *O. novo-ulmi* (MH75-4 O) were prepared as described previously^30^ and the spore density was adjusted to 10^7^ spore/ml based on initial densities determined with a hemocytometer.

### Inoculation

Saplings were maintained under standard greenhouse conditions (16 h light/24 °C and 8 h darkness/20 °C; and light intensity at 110 μmolm^−2^ s^−1^ (LI-250 A, LI-COR; Biosciences, Lincoln, NE, USA)) until mid-June. Fungal spores were inoculated into the growing trees by direct introduction of 0.01 ml spore suspension into the sap stream via a pre-drilled hole (standard steel drill bit, 1.5 × 40 mm). Inoculations with the fungus were carried out at many points along the main stem. Stem plugs (~2 cm^2^) around each inoculation point were collected at 0, 24, 48, 96, 122 and 144 h post-inoculation (hpi), immediately frozen in liquid nitrogen. Samples were stored at −80 ^o^ C until RNA extraction or hormone (SA and JA) quantification. The experiment was performed in triplicate. Due to extreme weather conditions, it was not possible to acquire the biological samples at 72 hpi.

### Scanning electron microscopy (SEM) of infected elm stems

Stem tissues 1 cm above the inoculation point were collected from the saplings of ‘Valley Forge’ and susceptible clone at 0, 48, 96 and 144 hpi. The samples were frozen in liquid nitrogen and stored in −80 ^o^C. Cross and longitudinal sections were prepared from frozen tissues using double sided razor blades. The sections were dried for 48 h in a vacuum desiccator, mounted on specimen stuns using sealing wax, sputter coated with gold using the K550x sputter coater (Quorum Technologies Ltd., Kent, UK) and then examined using a Hitachi S-570 Scanning Electron Microscope (Hitachi High Technologies, Tokyo, Japan).

### Investigations of the jasmonic acid (JA) and salicylic acid (SA) defence mechanism

To investigate the effect of defense elicitors on disease development, SA (2 mM) and MeJA (100 μM) were prepared in solutions containing 0.01% ethanol. Four-year-old American elm seedlings were purchased from a commercial nursery (Verbinnen’s Nursery Ltd, Dundas, ON, Canada) and were distributed in the field according to the randomized block design (Supplementary Fig. 7). The inoculation of the seedlings was carried out at a point on the main stem 20–25 cm above the soil surface and as described above. Seedlings were sprayed with 250 ml of SA, MeJA or 0.01% ethanol solutions using pressurized sprayers. Seedlings were well-maintained in the field and irrigated regularly to avoid any abiotic stress. At 60 days post-inoculation, disease was assessed based on the appearance of vascular coloration under the bark at the area 80 cm above the inoculation point. The experiment was performed in triplicate (n = 5) and the rate of disease incidence was calculated based on the number of seedlings showing brown streaking to the total number of inoculated seedlings. After observing disease symptoms, stem plugs (~ 2 cm^2^) were collected from the same area above the inoculation zone, frozen in liquid nitrogen and then stored in −80 ^o^ C until RNA extraction.

### RNA extraction and gene expression analysis

To examine the differential expression of disease-responsive genes, total RNA from elm stem samples was extracted using a modified CTAB protocol^64^. Briefly, 10 ml of CTAB extraction buffer (2% (w/v) CTAB (cetyl trimethylammonium bromide), 2% (w/v) PVP (polyvinylpyrrolidone, mol wt 40,000), 100 mM Tris-HCl (pH 8.0), 25 mM EDTA, 2 M NaCl, 0.05% spermidine trihydrochloride) was placed in sterile 50 ml conical centrifuge tubes and preheated to 65 ^o^C in a water bath for 30 min. Woody stem samples were ground in liquid nitrogen using IKA® A11 Basic Analytical Mill (IKA, Wilmington, USA), and further ground using a mortar and pestle. About 2 g of the ground tissues was quickly transferred to the warm extraction buffer after the addition of 2% of β-mercaptoethanol to the buffer. Tubes were vortexed vigorously for 2 min and then incubated at 65 °C for 45 min. Samples were vortexed every 15 min during incubation. Ten milliliter of chloroform-isoamyl alcohol (24:1) (Sigma-Aldrich Co., St Louis, USA) was added to each tube followed by vigorous vortexing for 2 min. The components of each tube were transferred to a clean Corex tube. Samples were then centrifuged at 26,000 × g for 10 min at 4 °C using Sorvall RC 6 plus (Thermo Electron Corporation, USA). The aqueous supernatant was transferred to a clean Corex tube and 1/3 volume of 8 M LiCl (Sigma) was added to the supernatant. Tubes were mixed well by shaking and then incubated for 2 h at −20 °C. The total RNA was pelleted by centrifugation at 26,000 × g for 30 min at 4 °C. The supernatant was discarded and the pellet was washed with 1 ml of 80% ethanol and centrifuged again at 26,000 × g for 10 min at 4 °C. The supernatant was removed and the pellet was dried at room temperature. The RNA pellet was dissolved in sterile Milli-Q water. The RNA quantity and quality was estimated using a Synergy H1 hybrid reader (BioTek, Oakville, ON, Canada) and 1.2% agarose gel containing 3% formaldehyde, respectively. All RNA extracts were treated with DNase using the RNase-free DNase set (Qiagen, Mississauga, ON) and then purified using RNeasy Mini Kit (Qiagen). The cDNA was synthesized from 2.5 μg of DNase treated RNA using the SuperScript® VILO™ cDNA Synthesis Kit (Invitrogen, Burlington, ON) in a total volume of 20 μl. Quantitative real time-PCR (qRT-PCR) was performed using CFX connect real-time Detection System (Bio-Rad, Mississauga, ON) and SsoFast™ EvaGreen® Supermix (Bio-Rad) using gene-specific primers (Supplementary Table 1). Results were statistically analyzed using the CFX manager software (Bio-Rad), which was also used to generate the Volcano plots and the clustergram.

### Analysis of cerato ulmin gene expression

To assess the transcriptional regulation of the fungal *cerato ulmin* (*CU*) gene by SA and MeJA treatments, transgenic fungal strains namely M75-LUC (L8) and M75-GUS (L4)^30^ were used. All fungal strains were initially grown on PDA media supplemented with 15 μg/ml hygromycin for seven days. To examine the induction of *LUC* by different treatments, 1 cm^2^ agar plugs were inoculated into 50 ml of liquid complete medium (CM)^65^ contained in 250 ml flasks and incubated for 7 days at 23 °C with agitation at 250 rpm. One milliliter of the 7d-old liquid culture was used to inoculate flasks containing 50 ml liquid medium supplemented with SA (1, 2, 4 mM), MeJA (25, 50, 100 μM) or 0.01% ethanol (control). Flasks were incubated at 23 °C with agitation at 250 rpm. Luciferase activity was measured every 24 h and for a total period of 144 h after treatment. To avoid changes in pH due to SA, the adjustment of pH was done after applying different concentrations of SA to the media. Both MeJA and 0.01% ethanol were added after autoclaving the media. To measure luciferase activity, 100 μl of the liquid medium was pipetted directly in the 96-wells microtitre plate and luciferase activity was measured using a Synergy H1 hybrid reader (BioTek), according to the method described previously^30^.

To examine the induction of *GUS* by defense elicitors, four agar plugs (~1 cm^2^) of M75-GUS (L4) fungal strain were transferred from 7d-old solid culture to a new PDA plate supplemented with 15 μg/ml hygromycin and incubated for three days at 23 °C. As a negative control, an agar plug form the M75-LUC fungal strain was transferred to each plate. Plates with growing mycelia were exposed to 5 ml of 0.01% ethanol (control), MeJA (50 μl) or SA (2 mM) and were left for 24 h at 23 °C before adding 10 ml of GUS staining solution to each plate^30^. Plates were kept at 37 °C overnight to develop the typical GUS color. After removing the GUS staining buffer, plates were photographed using a digital camera.

To investigate whether aggressive and non-aggressive fungal strains respond differently to plant defense elicitors (SA and MeJA), three strains of *O. ulmi* (H200-O, H956, Q412T-O) and *O. novo-ulmi* (VA-3 O, FG245-O, MH75-4 O)^66^,^67^ were exposed to 0.01% ethanol (control), MeJA (50 μM) or SA (2 mM) and the expression of *CU* gene was examined using qRT-PCR as described above. Briefly, a 7d-old liquid culture was prepared from each strain as described above and 1 ml of the liquid culture was used to inoculate 50 ml of liquid CM supplemented with defense elicitors. Flasks were incubated at 23 °C with agitation at 250 rpm for 122 h. The components of each flask were collected by centrifugation at 2,000 × g for 10 min. The supernatant was removed and the pellet was frozen in liquid nitrogen, ground using a mortar and pestle, and then used for RNA extraction and gene expression analysis the same way described above.

### Quantification of endogenous defense compounds

The levels of SA, JA and ferulic acid were determined in inoculated and control elm samples using previously published methods with minor modifications^68^,^69^. In brief, samples were accurately weighed and homogenized in acidified methanol (80% methanol, 20% 0.1 N trichloroacetic acid) in 1.5 mL Eppendorf tubes in a dark room with a Kontes Pellet Pestle disposable tissue grinder (Fisher Scientific) for 30 s. Homogenates were centrifuged (16,000 × g) for 3 min and the supernatant was filtered (0.2 μm, Ultrafree-MC filtered centrifuge tubes; Millipore; (16, 000 × g for 3 min). Salicylic acid (RT 2.66), ferulic acid (RT 2.91), and JA (RT 2.95) were separated from a 10 μL aliquot, at 30 °C, on a reverse phase column (150 × 2.1 mm, 1.7 μm C18 BEH, Waters Inc., Mississauga, ON) using a Waters Acquity I-Class UPLC. A gradient of 0.1% formic acid (Eluent A) and acetonitrile (Eluent B) [(A%:B%)]:0.0–05 min, 90:10; 0.5–3.5 min, 40:60; 3.5–4.2 min, 5:95; 4.2–6.5 min, 5:95; 6.5–7.0 min, 90:10] separated ferulic acid, SA and JA with a flow rate of 0.65 mL/min. Analytes were quantified with a tandem mass spectrometer (Xevo TQ-S triple quadrupole Mass Spectrometer, Waters). The capillary voltage was 3500, desolvation gas rate was 800 L/hr, cone gas rate was 150 L/hr, desolvation temperature was 400 °C, and the source temperature was 150 °C for all analyses with a dwell time of 0.02 s. Parent and daughter ions were detected using the appropriate MRM transitions and voltages (Supplementary Table 2). Standards were prepared at the following concentrations for each compound: 0, 5, 10, 50, 100, 250 and 500 ng/mL. The lower limit of quantification (LLOQ) was 1.0 ng/mL and the linear range was 10–250 ng/mL. The acquired data was processed with TargetLynx V4.1 (Waters Inc., Mississauga, ON).

### Statistical Analysis

One- or two-way analysis of variance (ANOVA) was performed using the ANOVA, GLM procedure of SAS statistical software (release 9; SAS Institute, Cary, NC). The statistical analysis of gene expression data was performed using the CFX manager software (Bio-Rad).

## Additional Information

**How to cite this article**: Sherif, S. M. *et al.* Simultaneous induction of jasmonic acid and disease-responsive genes signifies tolerance of American elm to Dutch elm disease. *Sci. Rep.* **6**, 21934; doi: 10.1038/srep21934 (2016).

## Supplementary Material

Supplementary Information
