# Supplementary material for: Simultaneous induction of jasmonic acid and disease-responsive genes signifies tolerance of American elm to Dutch elm disease
Source: Sci Rep. 2016 Feb 23;6:21934. doi: 10.1038/srep21934 (PMC4763294; doi:10.1038/srep21934)
Supplement: Supplementary Information [file srep21934-s1.pdf]

Simultaneous induction of jasmonic acid and disease-responsive genes signifies tolerance of American elm to Dutch elm disease

Sherif S.M[1,2]., Shukla M.R.[1], Murch S.J[3]., Bernier L.[4], Saxena P.K[1].

[1]Gosling Research Institute for Plant Preservation, Department of Plant Agriculture, University of Guelph, Guelph, ON, Canada

[2]Department of Horticulture, Faculty of Agriculture, Damanhour University, Al-Gomhuria St., PO Box 22516, Damanhour, Al-Behira, Egypt

[3]Chemistry Department, University of British Columbia, Kelowna, BC, Canada

[4]Centre d'étude de la forêt (CEF) and Institut de biologie intégrative et des systèmes (IBIS), Université Laval, Québec City, QC, Canada

SUPPORTING INFORMATION:

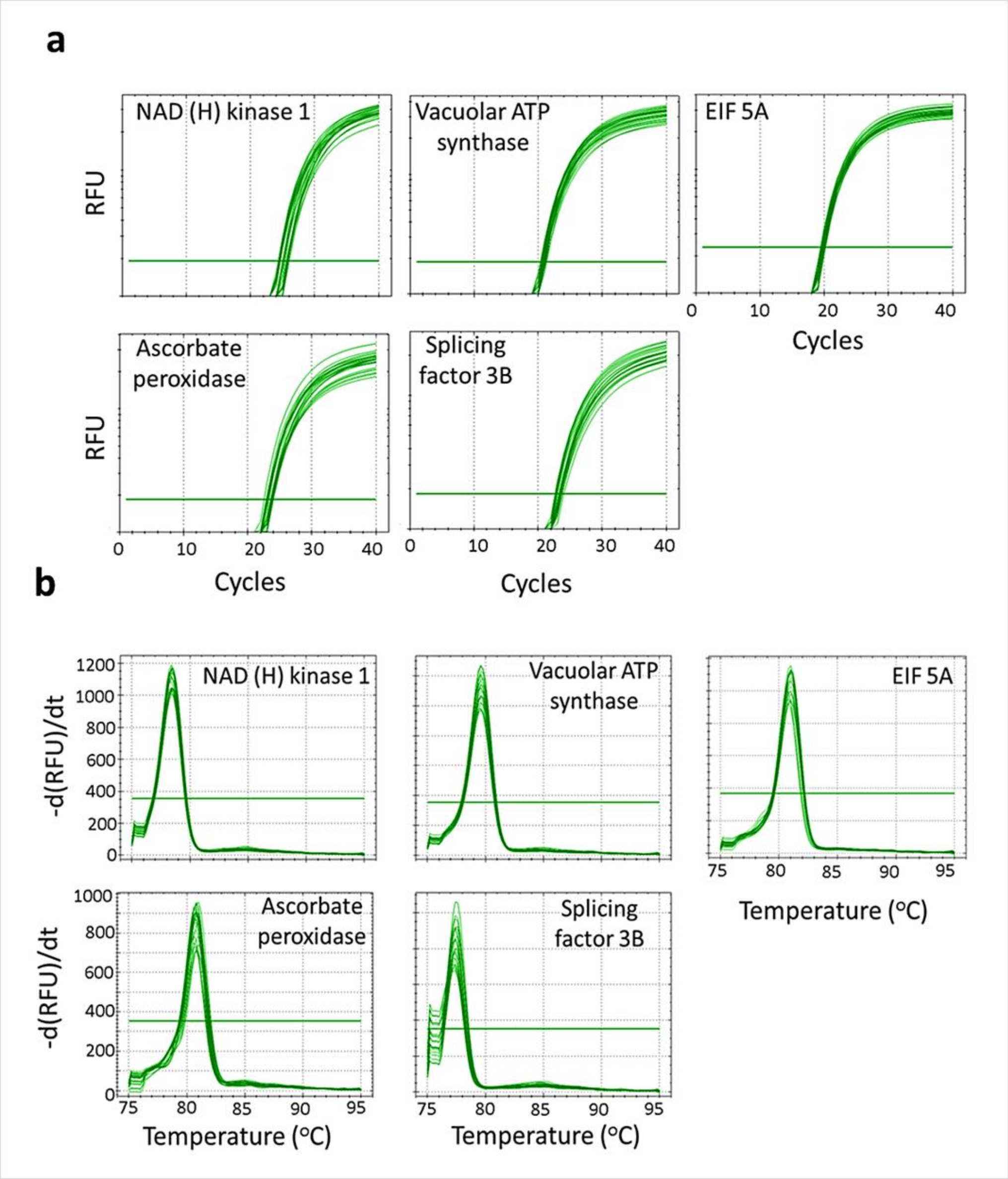

Supplementary Fig 1. The transcriptional stability and amplification characteristics of five internal reference genes investigated in elm saplings. (a) The amplification rate of five reference genes, encoding NAD (H) kinase 1, vacuolar ATP synthase, EIF 5a, ascorbate peroxidase and splicing factor 3B, in eight cDNAs (three technical replicates each) prepared from infected and non-infected ‘Valley Forge’ and susceptible elm saplings. The X-axis shows the number of PCR cycles while the Y-axis shows the relative fluorescence units (RFU). (**b)** Melt curve plots representing PCR products from the amplification shown in the upper panel.

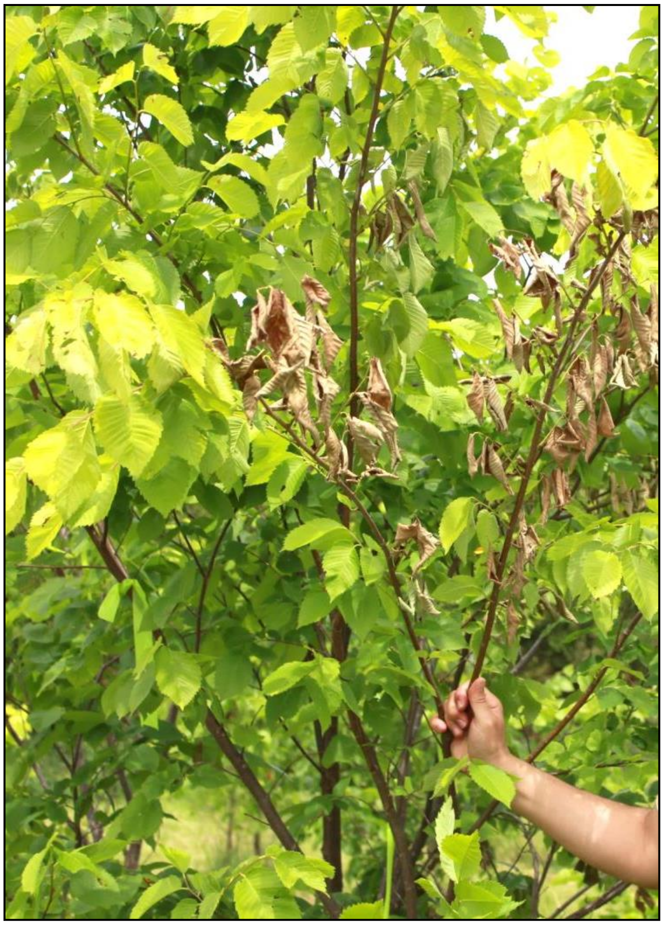

Supplementary Fig. 2: The aggressiveness of *Ophiostoma novo-ulmi* (MH75-4O) was examined in mature elm trees. Branches of a ten-year-old American elm tree grown were inoculated with 0.01 ml of spore suspension (10^7 spore/ml) and observed for disease symptoms after two weeks post-inoculation.

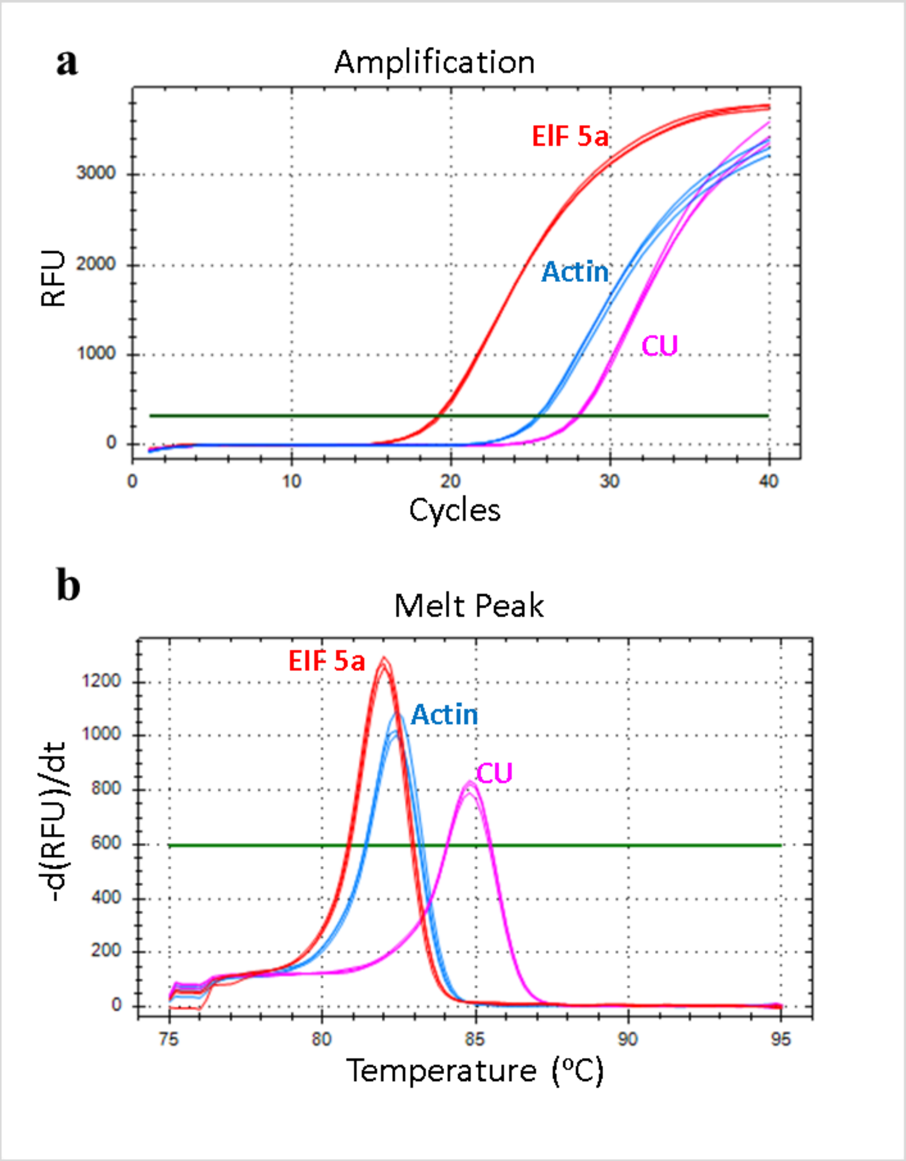

Supplementary Fig. 3: The amplification characteristics of elm and fungal genes investigated in infected elm wood tissues. (a) The expression of elm (*EIF 5a*) and fungal genes (*Actin*; *Cerato-ulmin*) was investigated in 'Valley Forge' and susceptible (s) elm saplings inoculated with the fungus for 0, 24, 48, 96, 122 and 144 hpi. The X-axis shows the number of PCR cycles while the Y-axis shows the relative fluorescence units (RFU). (**b**) Melt curve plots representing PCR products from the amplification shown in the upper panel.

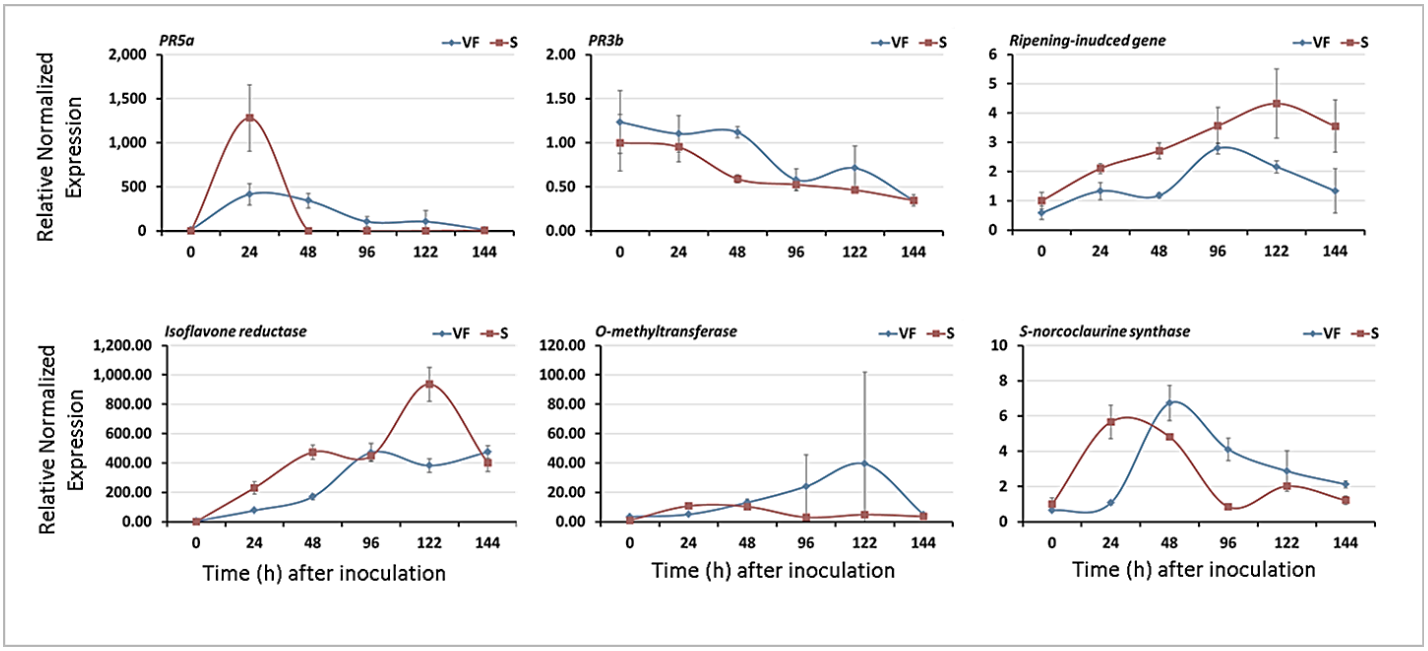

Supplementary Fig. 4: Differential expression of disease-responsive genes in tolerant and susceptible American elm saplings. The expression of six genes encoding for different classes of disease-inducible proteins was investigated in 'Valley Forge' (VF) and a susceptible (S) American elm clone at 0-144 hpi with the *O. novo-ulmi* fungus. The expression of each gene was normalized to that of three internal control genes (*EIF 5a, vacuolar ATP synthase* and *splicing factor 3B*) and was calculated relative to the control sample (0 hpi) in the susceptible clone. The results are the mean ± SE of three biological replicates.

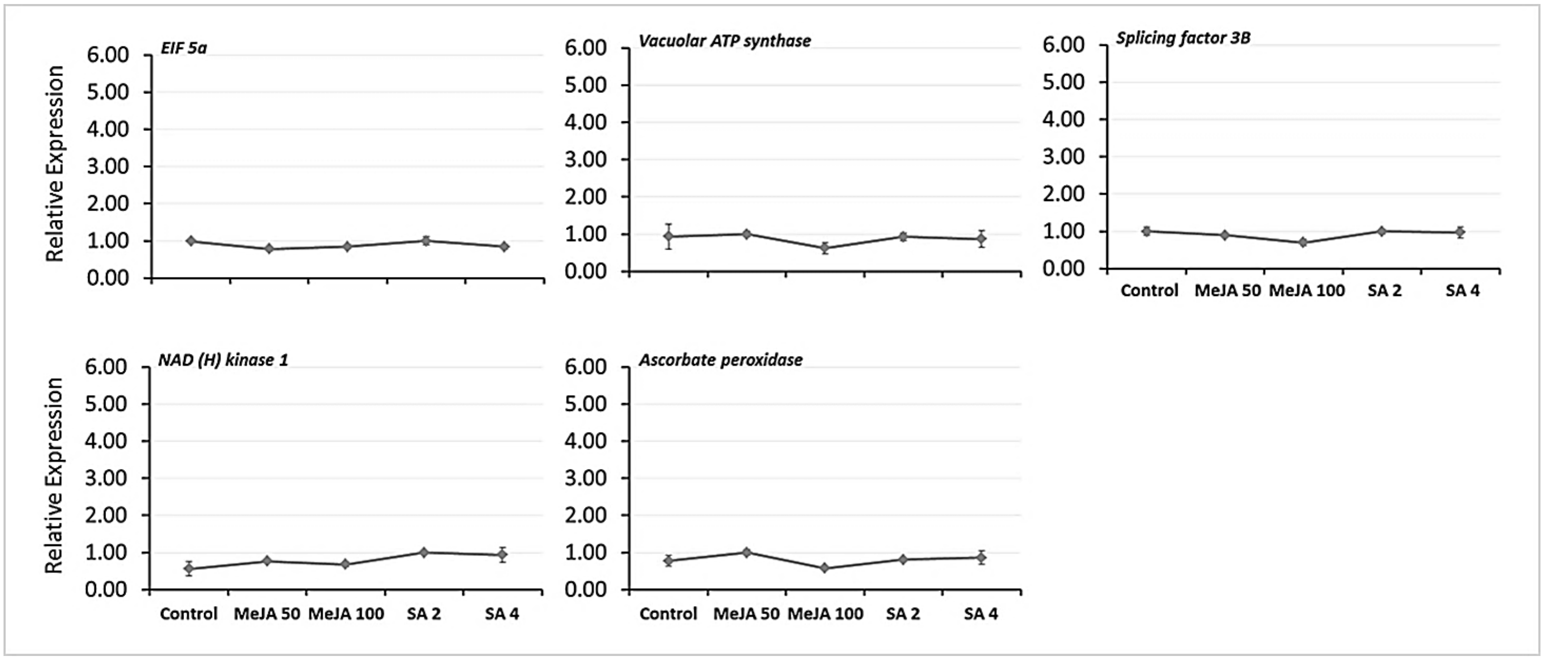

Supplementary Fig 5. The transcriptional stability of five reference genes after treatments with SA or MeJA. The expression of five internal genes encoding NAD (H) kinase 1, vacuolar ATP synthase, EIF 5a, ascorbate peroxidase and splicing factor 3B was investigated in the four-year-old saplings of the susceptible elm clone at 24h of treatment with SA (2mM), SA (4 mM), MeJA (50 µM) or MeJA (100 µM). The expression of each gene was calculated relative to the control sample (0.01% ethanol).

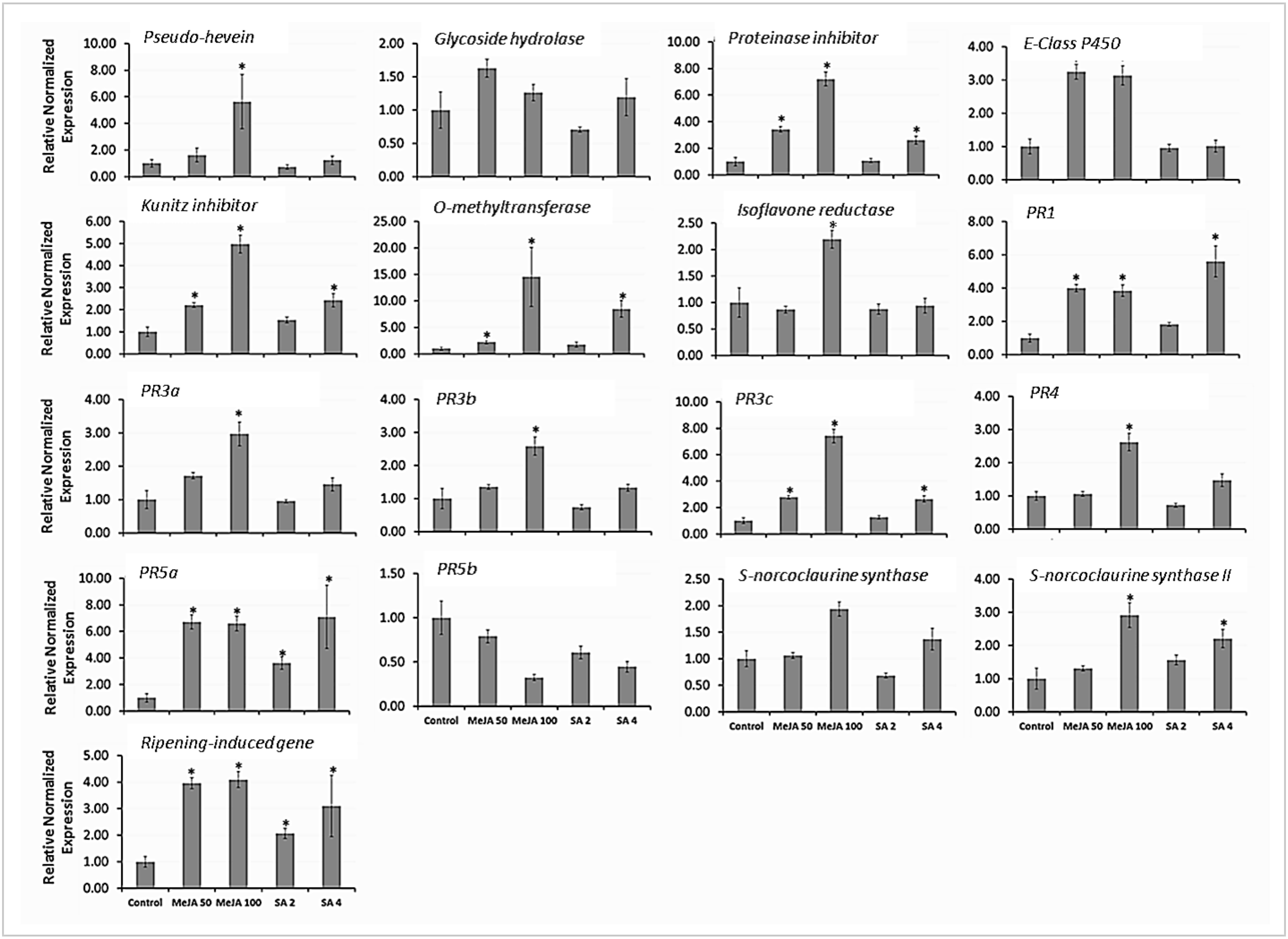

Supplementary Fig. 6: Differential expression of disease-responsive genes after treatments with SA or MeJA. The expression of six genes encoding for different classes of disease-inducible proteins were was investigated in the susceptible American elm clone at 24 hour of treatment with SA (2mM), SA (4 mM), MeJA (50 µM) or MeJA (100 µM). The expression of each gene was normalized to that of three reference genes (*EIF 5a*, *vacuolar ATP synthase* and *splicing factor 3B*) and was calculated relative to the control sample (0.01% ethanol). The results are the mean ± SE of three biological replicates.

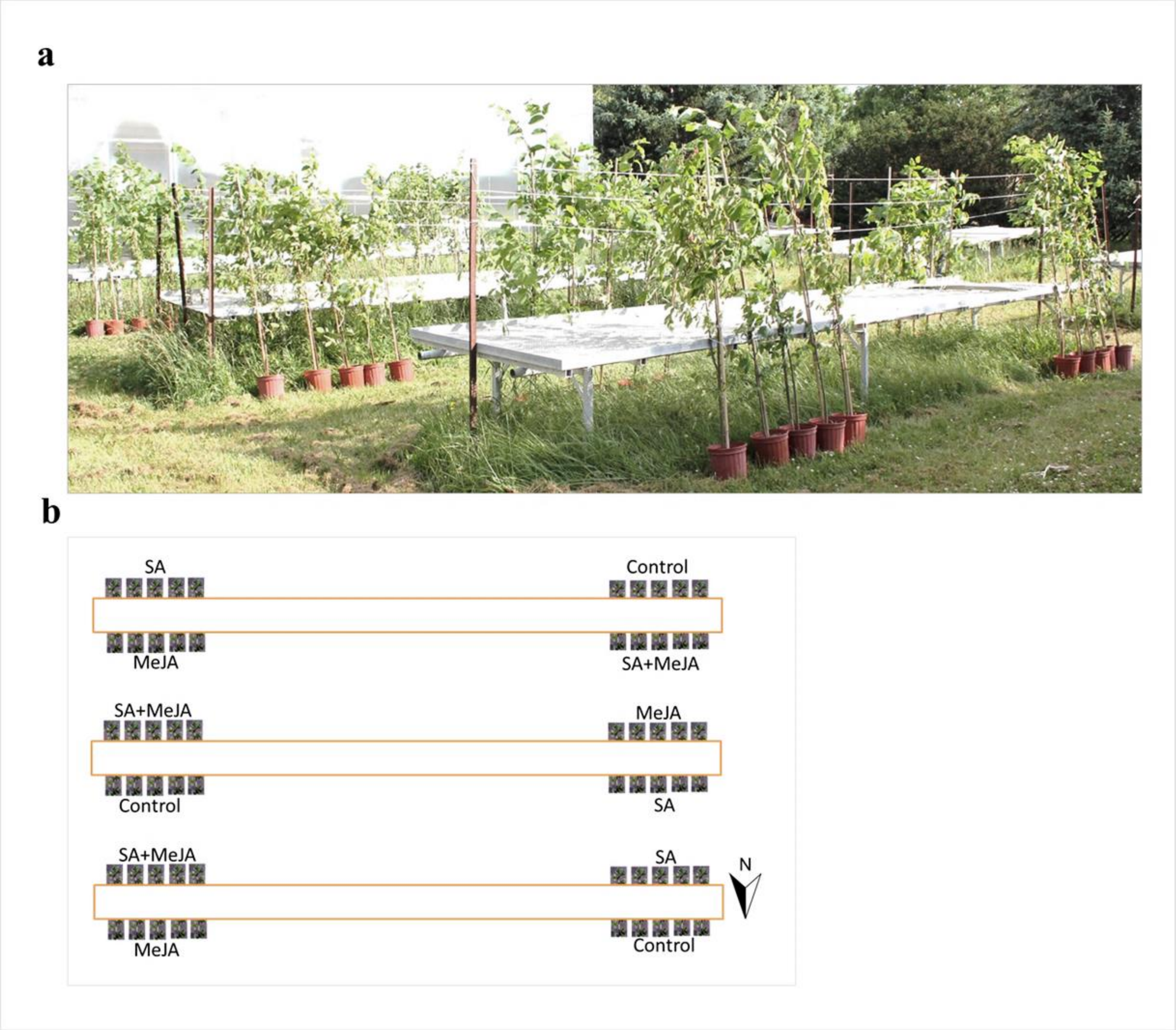

Supplementary Fig. 7: Distribution of American elm seedlings in the field according to the randomized block design. (a) Sixty American elm seedlings (4-year-old) distributed in an open field and used to examine the efficacy of applying defense elicitors to enhance the field tolerance of American elms to DED. (**b**) The distribution of treatments (Control, SA (2 mM), MeJA (50 µM) and SA (2 mM) +MeJA (50 µM) and replicates (three replicates (n=5)) in three field blocks. The arrow points to the North.

Supplementary Table 1: List of primer sequences used in this study.

Gene Name	Accession Number	Primer Sequence
ELF-F	FC325680	TCAAGGAGGGTTTTGCTGA
ELF-R		CCAACGTCCTTGAGACCAC
Pseudo-hevein-F	FC325206	GACAAGCCCTACGCTTTCC
Pseudo-hevein-R		GGCACTTGCCACAAGACTC
Glycoside hydrolase-F	FC325261	TGCCAATATGCTGGAAACC
Glycoside hydrolase-R		GAAGCCCCAAGAACACCTG
E-class P450-F	FC325322	TTACTACGCCTGGGAATGC
E-class P450-R		AAGCGAGGCTTGATGAGAA
Isoflavone reductase-F	FC325532	TCCGGACTTGTTGAAGCTC
Isoflavone reductase-R		GGACATCCCACATTTGCTC
O-methyltransferase-F	FC325422	CCCTCTGATACAGGCATCG
O-methyltransferase-R		CACATATCCTTGCCGGTTG
S-norcoclaurine synthase-II-F	FC325579	GGCGTTGGCACTATTGTCT
S-norcoclaurine synthase-II-R		TCATGGTCGATCTTGGTGA
Ripening-induced gene-F	FC325592	TGCTTCGCCAACTGTATCA
Ripening-induced gene-R		AGCTTAGGGGCTGATTTGG
Kunitz inhibitor-F	FC325621	TCGCAGGGATCATTATCGT
Kunitz inhibitor-R		TCTCGGTGACCTTTGATCC
S-norcoclaurine synthase-F	FC325445	CCACCTCCATTGCTTTCAC
S-norcoclaurine synthase-R		TCTGCCTCAAATTCGTTCC
Proteinase inhibitor-F	FC325589	TGCAGTGGAGACAATCGAG
Proteinase inhibitor-R		CCAAACCCTTGAGCAGTCA
PAL-F	DQ078279	GCTCTTAACAACGGCGAAA
PAL-R		GGGCAAAAGGGTCTTCAAT
PR5b-F	FC325266	CTGTAAGCTTCCGGGCATT
PR5b-R		TGCACTGGTGCGTTTAACA
PR5a-F	FC325601	CCGCAATGACTGCCATTAT

PR5a-R		CCACCCAAAGACTCCAGGT
PR4-F	FC325240	CTGGGACCTGAACAGAGCA
PR4-R		CAAAAGGCAGTCCATCCAA
PR3a-F	FC325284	CCCCGGCAAGAGTTTCTAT
PR3a-R		CCCGTTTAGAAGCCTCAGC
PR1-F	FC325238	TCGTGATCAGGTGGGTGTT
PR1-R		TACAGTCGCCTTTCCGTTG
PR3b-F	FC325581	TCCATGCAACCCTAGCAAA
PR3b-R		CGAAGTTGAGGGCTTCTCC
PR3c-F	FC325338	TGAGGCTTCTAAACGGGAGA
PR3c-R		CGCCTCCCGTCTCATCTAT
Vacuolar ATP synthase-F	FC325256	GCTGCAGAGCAAGAAGCTC
Vacuolar ATP synthase-R		CAGCCTCTTCTTTGGCTTG
Splicing factor 3B-F	FC325436	TTCCCCCTGAAAGAGAGGA
Splicing factor 3B-R		CAACCAACCGGATTTTCAG
NAD (H) kinase 1-F	FC325344	TCGTCACATGTGTCCAAGG
NAD (H) kinase 1-R		AACCATTGATCCTCCAGCA
Ascorbate peroxidase-F	FC325348	CACCACATCTTCGGGACAT
Ascorbate peroxidase-R		ATGTGCCCTTCCCAGTGTA
On-Actin-F	AF378562	ATCAACCCCAAGTCCAACC
On-Actin-R		GGCCTGGATGTTGACGTAG
On-Cerato ulmin-F	KF725663	AGCAACAGCGACTCCTACG
On-Cerato ulmin-R		AAGATTGGCCACACCAAGA

Supplementary Table 2: Mass Spectrometer Parameters

**Compound**	**Formula**	**Parent Ion (m/z)**	**Daughter Ion (m/z)**	**Cone (V)**	**Collision Cell (eV)**
Jasmonic Acid	$C_{12}H_{18}O_3$	211.13	133	22	14
Jasmonic Acid	$C_{12}H_{18}O_3$	211.13	151	22	10
Ferulic Acid	$C_{10}H_{10}O_4$	195.18	167	26	10
Ferulic Acid	$C_{10}H_{10}O_4$	195.18	95	26	18
Salicylic Acid	$C_7H_6O_3$	139.12	121	6	12
Salicylic Acid	$C_7H_6O_3$	139.12	95	6	10
